# Abundance and associated factors of *Amblyomma tigrinum* (Acari: Ixodidae) infesting wild foxes in north-central Chile

**DOI:** 10.1590/S1984-29612023062

**Published:** 2023-10-27

**Authors:** Felipe Hernández, Jonatan Manqui, Daniel González-Acuña, Esperanza Beltrami, Claudio Verdugo, Gerardo Acosta-Jamett

**Affiliations:** 1 Instituto de Medicina Preventiva Veterinaria, Facultad de Ciencias Veterinarias, Universidad Austral de Chile, Valdivia, Chile; 2 Center for Surveillance and Evolution of Infectious Diseases, Facultad de Ciencias Veterinarias, Universidad Austral de Chile, Valdivia, Chile; 3 Programa de Magíster en Ecología Aplicada, Facultad de Ciencias, Universidad Austral de Chile, Valdivia, Chile; 4 Facultad de Ciencias Veterinarias, Universidad de Concepción, Chillán, Chile; 5 Instituto de Patología Animal, Facultad de Ciencias Veterinarias, Universidad Austral de Chile, Valdivia, Chile

**Keywords:** Amblyomma, wild carnivores, Southern Cone of America, tick loads, local-scale factors, Amblyomma, carnívoros silvestres, Cone Sul da América, cargas de carrapatos, fatores de escala local

## Abstract

The tick *Amblyomma tigrinum* inhabits areas with diverse climatic conditions, with adult stages parasitizing wild canids, such as chilla (*Lycalopex griseus*) and culpeo (*Lycalopex culpaeus*) foxes. We described the infestation loads in wild foxes captured at three sites (periurban, rural and wild) through an anthropization gradient in north-central Chile. We tested whether local-scale environmental and/or individual host factors can predict tick abundance by using negative binomial models. During 2018-2020 (spring and summer), we captured 116 foxes (44 chillas and 72 culpeos), and 102 of them were infested with ticks (87.9%, CI=80.6-93.2%). We collected 996 *A. tigrinum* adult ticks, estimating a total mean abundance of 8.6±0.8 ticks/host. Periurban and rural foxes harbored greater tick loads than foxes from the wild site (2.34 and 1.71 greater, respectively) while tick abundance in summer decreased by up to 57% compared to spring. Tempered, more humid climate conditions of the periurban site could favor the development and survival of adults *A. tigrinum*; and ticks may have adopted a quiescent stage or similar survival mechanisms to cope with summer temperature increases related to the ongoing megadrought. Further studies are warranted to understand the underlying factors determining the life cycle of *A. tigrinum* at larger spatiotemporal scales.

## Resumo

*Amblyomma tigrinum* habita em áreas com diversas condições climáticas, com estágios adultos, geralmente, parasitando canídeos silvestres, como chilla (*Lycalopex griseus*) e culpeo (*Lycalopex culpaeus*). Neste trabalho, são descritas as cargas de infestação de *A. tigrinum* em raposas silvestres, capturadas em três locais (periurbano, rural e silvestre) no centro-norte do Chile. Foi testado se os fatores ambientais e/ou individuais do hospedeiro, em escala local, podem prever a abundância de carrapatos, usando-se modelos binomiais negativos. Durante 2018-2020 (primavera e verão), foram capturadas 116 raposas (44 chillas e 72 culpeos), e 102 delas estavam infestadas por carrapatos (87,9%, IC=80,6-93,2%). Foram coletados 996 carrapatos adultos de *A. tigrinum*, estimando-se uma abundância média total de 8,6±0,8 carrapatos/hospedeiro. As raposas periurbanas e rurais abrigaram cargas de carrapatos maiores do que as raposas do local silvestre (2,34 e 1,71 maior, respectivamente), enquanto a abundância de carrapatos, no verão, diminuiu até 57% em comparação com a primavera. As condições climáticas temperadas e mais úmidas do sítio periurbano podem favorecer o desenvolvimento e a sobrevivência dos adultos de *A. tigrinum*; e os carrapatos podem ter adotado um estágio quiescente ou mecanismos de sobrevivência semelhantes, para lidar com os aumentos de temperatura no verão, relacionados à mega seca em andamento.

*Amblyomma* (Acari: Ixodidae) is a tick genus represented by large-sized ornamented ticks, with approximately 25 species exhibiting a wide geographical distribution through Southern Cone of America (Nava et al., 2017). For instance, the tick species *A. tigrinum* has been recorded to thrive across areas with contrasting climatic conditions such as lowlands in central Chile (i.e., from the Coquimbo region to the Aysén region) (Mastropaolo et al., 2008; Abarca et al., 2016) and all phytogeographic regions in continental Argentina (Guglielmone et al., 2000), among other locations. Adult stages of *A. tigrinum* are described as usually parasitizing domestic and wild members of the Canidae family (Abarca et al., 2016) and occasionally other carnivore hosts (e.g., Manqui et al., 2022); while immature stages can parasitize small rodents (Cricetidae and Caviidae) and wild birds (Nava et al., 2017; Guglielmone et al., 2021). In south-central Chile, this tick regularly infests free-ranging foxes, such as chillas (*Lycalopex griseus*) and culpeos (*Lycalopex culpaeus*) (Cevidanes et al., 2021; González-Acuña et al., 2003). However, there is a paucity of data about the ecology of *A. tigrinum* in fox populations at their northern native range in the country. Our research aimed to further investigate the presence and infestation patterns of *A. tigrinum* retrieved from chillas and culpeos inhabiting a wildlife-domestic interface of north-central Chile, and explore whether local-scale environmental and/or individual host factors can predict tick abundance.

**Figure 1:**
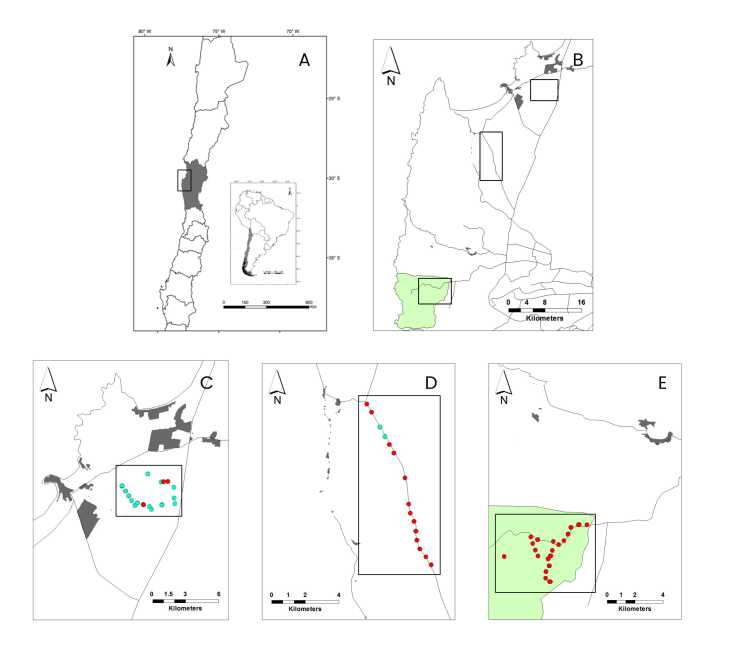
Map of wild fox captures in the coastal zone of the Coquimbo region, Chile. *Top left*: (A) Black rectangular contour denotes the study area in the Coquimbo region (gray area). *Top right*: (B) Black rectangular contours denote the three field sites. Bosque Fray Jorge National Park (BFJNP; green area) is indicated. *Bottom*: (C) Periurban, (D) Rural, and (E) Wild sites. Light blue and red dots indicate captures of chilla and culpeo foxes, respectively. Proximate human settlements (gray polygons) and main roads (black lines) are drawn around each site.

The study was conducted in the coastal zone of the Coquimbo region in north-central Chile (71°12’ to 71°40’W, 29°58’ to 30°39’S). The study area poses a semiarid Mediterranean weather with a mean annual rainfall of 126.8mm, with 90% of rainfall concentrated during winter months (May-September), and warm, dry summers (December-March) (Montecinos et al., 2016). Mean temperature ranges from 12 to 18ºC (measured at 2 m above ground nearby the coast), and relative air humidity can reach 90 to 100% at higher altitudes (ranges from 100 to 300 m across the study area). We defined three field sites running from the coastal cities of Tongoy and Guanaqueros through areas of decreasing human perturbation to Bosque Fray Jorge National Park (BFJNP), which were categorized as Periurban, Rural and Wild, respectively (see Hernández et al., 2021 for details) (Figure 1).

During September 2018 – March 2019 and September 2019 – February 2020, we surveyed chillas and culpeos within all field sites, which were captured with leg-hold traps (Victor Soft Catch No. 1.5, Chagnons Trapping Supply, Manistique, Michigan, USA; size 13 cm of diameter). A total of 20 traps baited with canned jack mackerel fish and wolf urine (Murray’s Lure & Trapping Supplies, USA) were set up during 7 to 10 consecutive nights at each site. Trapping was conducted for a total of 1,860 trap-nights (average 620 trap-nights per site). Foxes were anesthetized with a ketamine-dexmedetomidine association, and reversed with atipamezole (modified from Acosta-Jamett et al., 2010). While anesthetized, we inspected each animal’s body coat and collected ticks for ten minutes. All foxes were marked with unique subcutaneous microchips and were safely released at the site of capture.

Individual ticks were counted and examined morphologically under a stereomicroscope, according to the keys of Estrada-Peña et al. (2005) and Nava et al. (2017). We estimated the prevalence (95%CI) and mean abundance of *A. tigrinum* in the sampled wild foxes (following Bush et al., 1997). To assess and host individual factors that may be associated with tick abundance, we considered five variables corresponding to: (a) site (periurban, rural and wild); (b) season (spring and summer combined between both sampling periods); and (c) species, (d) sex, and (e) age of sampled foxes. Given tick total counts were over-dispersed (previously tested by using the sum squared Pearson residuals divided by residuals degrees of freedom), we specified models with a negative binomial distribution for all the following statistical analyses. We used generalized linear models to analyze the relationship between the tick loads with the defined environmental and host-dependent variables. We carried out the models using the glm.nb function from the R-package MASS (Venables & Ripley, 2002). For model selection, we computed and ranked models by AIC criteria corrected for small sample size (AIC_c_) using the R-package MuMIn (Barton, 2017), and reported the incidence rate ratio (IRR) for each predictor in the most supported model with ΔAIC_c_<2. Data were analyzed using R version 4.1.0 software, and *p*<0.05 was considered statistically significant. Recaptured foxes (*n*=5) were considered independent observations because there were at least three months of difference between captures, and then, we considered that in that period the foxes had the chance to become re-infested (following Cevidanes et al., 2021).

Overall, we captured 116 foxes (44 chillas and 72 culpeos), with 102 out of 116 (87.9%, CI=80.6-93.2%) individuals exhibiting tick infestation. We identified all 996 collected ticks as adults of the species *A. tigrinum* (561 males and 435 females), resulting in a mean abundance of 8.6±0.8 ticks per host (range 0-55). Foxes sampled at the periurban site harbored 467 ticks (mean abundance 11.7±1.6), while foxes sampled at the rural and wild sites were infested by 269 (mean abundance 9±1.5) and 260 (mean abundance 5.7±1.0) ticks, respectively. Foxes captured during spring harbored 647 ticks (mean abundance 11.8±1.4), while foxes sampled in summer exhibited 349 ticks (mean abundance 5.7±0.7) (Table 1). Environmental factors such as site and sampling season significantly predicted the abundance of *A. tigrinum* harbored by wild foxes, and both variables were included in the three best-ranked AIC_c_ models, totaling a cumulative AIC_c_ weight of evidence of 0.88 (data not shown). Based on the top-ranked model, the tick load of periurban foxes was 2.34 times greater than foxes from the wild site while holding all other variables constant; while tick infestation in rural foxes resulted in 1.71 greater than foxes from the wild site (Table 2). During summer, tick loads of foxes decreased by 57% compared to spring (Table 2). Other variables such as fox species, sex or age did not significantly predict variations of *A. tigrinum* infestation across field sites and seasons (IRR 95%IC included 1).

**Table 1:** Prevalence and mean abundance of *Amblyomma tigrinum* ticks retrieved from wild foxes per field site and season, from September 2018 to February 2020, in the coastal zone of the Coquimbo region, Chile.

	No. foxes	No. ticks	Tick sex		Prevalence (95% CI)^a^	Mean abundance (±S.E.)^b^
			Male	Female		
Site						
Periurban	40	467	276	191	92.5 (79.6-98.4)	11.7 ± 1.6
Rural	30	269	140	129	96.7 (82.8-99.9)	9 ± 1.5
Wild	46	260	145	115	78.3 (63.6-89.1)	5.7 ± 1.0
Season						
Spring	55	647	362	285	92.7 (82.4-98.0)	11.8 ± 1.4
Summer	61	349	199	150	83.6 (71.9-91.8)	5.7 ± 0.7
Total	116	996	561	435	87.9 (80.6-93.2)	8.6 ± 0.8

a Confidence intervals;

b Standard error.

**Table 2:** Estimates and incidence ratios (IRR) of predictor variables related to the abundance of ***Amblyomma tigrinum* of wild foxes from Coquimbo region, Chile. Parameters values of the best-ranked** AIC_c_ model are presented.

	Estimate (SE)	IRR	IRR 95% CI	*z*-value^d^	Pr (>|*z*|)^e^
Rural site^a^	0.54 (0.23)	1.71	(1.11-2.68)	2.38	0.02*
Periurban site^a^	0.85 (0.21)	2.34	(1.55-3.52)	4.12	0.00*
Summer^b^	-0.85 (0.18)	0.43	(0.30-0.61)	-4.77	0.00*
Female^c^	0.27 (0.18)	1.31	(0.92-1.86)	1.49	0.14

* *p* < 0.05.

a Wild site defined as reference site;

b Spring defined as reference season;

c Male defined as reference fox sex;

d *z* test statistic;

e *p*-value associated to *z* test statistic.

Our study constitutes the first report of *A. tigrinum* infesting chilla and culpeo foxes in the coastal area of the Coquimbo region. We found higher tick loads harbored by foxes inhabiting nearby a human-altered site compared to more distant rural sites across a coastal anthropization gradient. On the other hand, foxes sampled during spring exhibited higher tick infestations than foxes sampled during summer. Site and seasonal-related factors associated with *A. tigrinum* abundance may respond to underlying bioclimatic features such as fluctuations in temperature and relative humidity, which have been implied in the habitat suitability for other *Amblyomma* species (e.g., Clarke-Crespo et al., 2020). Data derived from a parallel study on small terrestrial mammals within the study area indicated that mean temperatures measured at the periurban site (range: 15.8-17.3°C) were about two and three centigrade lower than the wild (range: 17.7-19.3°C) and rural (range: 18.4-20.1°C) sites, respectively; while the mean relative humidity at the periurban site (range: 68.3-72.1%) was almost six percent higher than the rural site (range: 62.8-66.6%) (Beltrami, 2021). Due to proximity to the coastal border, tempered, more humid climate conditions of the periurban zone could protect ticks from desiccation, thus, favoring the development and survival of adult *A. tigrinum* specimens (Pfäffle et al., 2013).

In north-central Chile, a megadrought occurring from 2010 to the present has worsened both bioclimatic conditions and vegetational productivity at the microhabitat level (Garreaud et al., 2017, 2020). Then, the higher infestation of *A. tigrinum* on foxes during the spring season could be partially related to extreme climatic conditions of summer periods associated with the megadrought. While all parasitic stages of *A. tigrinum* are active throughout the year, they exhibit higher activity from late spring to early (adults) or middle (larvae and nymphs) fall in temperate climates (Guglielmone et al., 2000; Nava et al., 2009). However, their life cycle appears to be further regulated by temperature fluctuations, and when facing unfavorable environmental conditions, *Amblyomma* ticks can become quiescent until conditions become more favorable. This is a non-stational nor programmed survival strategy where ticks (and other arthropods) exhibit slowed metabolism and reduce their activity triggered by acyclic environmental changes characterized by high temperature conditions (Diniz et al., 2017; Nava et al., 2009*)*. In general, during our survey, the summer period was warmer (range: 19.0-20.6°C) than the spring period (range: 15.6-17.3°C) (Beltrami, 2021); thus, perhaps ticks may have adopted a quiescent stage or similar survival mechanisms to cope with seasonal temperature increases.

On the other hand, we cannot rule out that the presence of other suitable *A. tigrinum* hosts may contribute to maintaining a higher abundance of ticks in the periurban site compared to the rural and wild sites. For example, domestic dogs interact indirectly with free-ranging foxes within the Tongoy-Guanaqueros urban periphery (Acosta-Jamett et al., 2011; Hernández et al., 2021). *A. tigrinum* had been found infesting dogs from rural areas in Coquimbo and La Araucanía region (Abarca et al., 2016). These findings are consistent with reports of adult *A. tigrinum* that successfully fed on dogs under laboratory conditions (Labruna et al., 2002). Other carnivores such as the Molina’s hog-nosed skunk (*Conepatus chinga*) have been reported to harbor *A. tigrinum* and this mephitid co-occurs with foxes across this zone (Manqui et al., 2022); therefore, there may be potential cross-species tick infestation from environments domestic/wild carnivores share. One of the limitations of our study is that we did not assess the abundance and infestation of wild birds with immature stages in the different areas and seasons (Labruna et al., 2002), which could also potentially contribute to the spatiotemporal differences observed in our study. Evaluating the presence and involvement of wild birds in the life cycle of the studied ticks could provide valuable insights into the overall dynamics of tick infestation and transmission pattern.

As a whole, our study contributes to enhancing the knowledge about the local-scale environmental factors that determine the abundance of *A. tigrinum*, which may play a certain role as a vector of zoonotic infectious agents (Abarca et al., 2013; Cicuttin et al., 2017). Further studies are warranted to understand the underlying factors determining the life cycle of *A. tigrinum* at larger spatiotemporal scales, including systematic assessments of intermediate hosts (i.e., small rodents and wild birds) for tick maintenance through human-altered landscapes.
